# Molecular Testing in Thyroid Nodules: How Much Does It Change Clinical Practice?

**DOI:** 10.3390/biomedicines13081947

**Published:** 2025-08-09

**Authors:** Mehmet Kostek, Niranjna Swaminathan, Azeem Izhar, Andrea Gillis, Herbert Chen, Brenessa Lindeman

**Affiliations:** Department of Surgery, University of Alabama at Birmingham, Birmingham, AL 35233, USA; nswaminathan@uabmc.edu (N.S.); aizhar@uabmc.edu (A.I.); agillis@uabmc.edu (A.G.); hchen@uabmc.edu (H.C.); blindeman@uabmc.edu (B.L.)

**Keywords:** fine needle aspiration biopsy, molecular testing, thyroid cancer, thyroidectomy, thyroid nodule

## Abstract

**Background/Objectives:** Molecular testing is most commonly performed in evaluation of thyroid nodules with indeterminate Fine Needle Aspiration Biopsy (FNAB) results. However, in clinical practice, thyroidectomy may still be pursued in patients who present with clear clinical indications despite a benign molecular test result. The aim of this study is to identify clinical factors that influence the decision to proceed with surgery in the presence of a benign molecular test result. **Methods:** Patients who were evaluated in the outpatient clinic for thyroid nodules at one institution between January 2016 and January 2024 were retrospectively reviewed. Patients with FNAB results corresponding to Bethesda categories III or IV and a benign result on the Afirma molecular test were included. Demographic data, medical and family history, characteristics of thyroid nodules (including ultrasonographic features), surgical history, and postoperative pathology results were analyzed. Patients were divided into two groups based on clinical management—Observation (Group-1) or Thyroidectomy (Group-2)—and compared using Chi-square tests for bivariate analysis and multivariable logistic regression. **Results:** A total of 177 patients were included, with 87 (49.1%) in the observation group and 90 (50.9%) in the surgical group. Mean age was 55.9 ± 13.9 years and median nodule size (IQR) was 2.8 cm (1.95–4.0 cm). Bivariate analysis revealed the surgical group had significantly higher proportions of patients with compressive symptoms (*p* < 0.001), hyperthyroidism (*p* = 0.01), nodules >4 cm (*p* < 0.001) and documented nodule growth during follow-up (*p* < 0.001). Multivariate logistic regression identified the following factors as independently associated with the decision to proceed with surgery: compressive symptoms (OR: 23.2; 95%CI: 6.06–88.89; *p* < 0.001), hyperthyroidism (OR: 5.87; 95%CI: 1.63–21.20; *p* = 0.007), nodule size >4 cm (OR: 11.36; 95%CI: 3.90–33.12; *p* < 0.001), and increasing nodule size during follow-up (OR: 7.85; 95%CI: 2.72–22.65; *p* < 0.001). **Conclusions:** Despite a benign molecular test result, patients exhibiting compressive symptoms, hyperthyroidism, nodules larger than 4 cm, or evidence of nodule growth during follow-up are significantly more likely to undergo thyroidectomy. In such cases, molecular testing may offer limited clinical utility and could be omitted to optimize cost-effectiveness.

## 1. Introduction

The global incidence of differentiated thyroid cancer has been steadily increasing [1]. Diagnostic evaluation of thyroid cancer typically begins with the evaluation of thyroid nodules with ultrasonography, which provides critical information on the composition, echogenicity, shape, margin and the presence of calcifications [2]. Nodules with suspicious sonographic features for malignancy are then typically sampled with Fine Needle Aspiration Biopsy (FNAB) [3]. FNAB results are interpreted and reported using the Bethesda System for Reporting Thyroid Cytopathology, which classifies findings into six categories. Among these, three categories—Atypia of Undetermined Significance (AUS), Follicular Neoplasm (FN) and Suspicious for Malignancy (SFM)—are considered indeterminate, meaning they do not definitively indicate benign or malignant pathology [4]. These indeterminate categories account for approximately 15–30% of all FNAB results [5]. While SFM is associated with a high estimated risk of malignancy (up to 74%), often leading to thyroidectomy, the management of nodules categorized as AUS or FN remains nuanced and frequently relies on a combination of clinical judgment and patient preferences [6,7].

Molecular testing has emerged as a useful adjunct for the management of indeterminate thyroid nodules, alongside other strategies such as repeat biopsy, surgical intervention, and active surveillance [3,8,9]. These tests identify specific genetic alterations associated with thyroid cancer, thereby offering additional insights into the likelihood of malignancy [10]. A positive molecular test result typically supports the decision to proceed with surgery. However, when the molecular test result is benign, clinical decision-making becomes more complex. Furthermore, indiscriminate use of molecular testing in cases where clinical features already suggest surgical management may contribute to unnecessary healthcare expenditures [11].

To enhance cost-effectiveness and streamline care, it is critical to determine whether a benign molecular test result in the context of an indeterminate FNAB meaningfully alters clinical management, as numerous factors beyond molecular findings influence whether to pursue surgery in clinical practice. The objective of this study is to identify the clinical factors that lead to surgical management of indeterminate thyroid nodules despite benign molecular test results in order to determine which patients do not require additional sampling for molecular testing during FNAB.

## 2. Methods

Following approval from the Institutional Review Board, a retrospective review was conducted of patients evaluated for thyroid nodules in the outpatient clinic of a single center between January 2016 and January 2024. Patients were eligible for inclusion if they had indeterminate FNAB results, specifically Atypia of Undetermined Significance (AUS) or Follicular Neoplasm (FN), and a benign result on the Afirma Gene Expression Classifier (GEC) or Gene Sequencing Classifier (GSC) (Veracyte, Inc., South San Francisco, CA, USA). Patients who were lost to follow up before receiving molecular test results or those with an ASA (American Society of Anesthesiologists) Class of 4 or higher which could influence management decisions were excluded from the study.

The following data were extracted and analyzed: demographic characteristics (age, gender, race, ethnicity), weight, Body Mass Index (BMI), clinical symptoms (dyspnea, dysphagia, voice changes, globus sensation), past medical and family history, thyroid nodule sonographic features including ACR-TIRADS score, FNAB results, surgical history, and postoperative pathology results. Patients were divided into two groups according to clinical management decision: Observation (Group 1) or Thyroidectomy (Group 2).

Compressive symptoms were defined as the presence of dysphagia, dyspnea, hoarseness and/or a sensation of neck fullness. A history of radiation exposure was defined as prior neck radiotherapy or exposure nuclear events. Subclinical and clinical hypothyroidism were grouped together under the diagnosis of hypothyroidism; similarly, subclinical and clinical hyperthyroidism were considered collectively as hyperthyroidism. A positive family history of thyroid disease was defined as any first-degree relative with thyroid cancer or a diagnosis of hypo—or hyperthyroidism. Nodule growth during follow up was defined as a >20% increase in at least two dimensions of the nodule in one year interval between two ultrasound examination.

The normality of continuous variables was assessed using the Shapiro–Wilk test. Age was found to be normally distributed so is presented as mean ± standard deviation, while non-normally distributed variables are reported as median with interquartile range (IQR). For statistical comparisons, Student’s *t*-test was used for normally distributed continuous variables, and the Mann–Whitney U-test was applied to non-normally distributed data. Categorical variables were compared using the Chi-square test or Fisher’s exact test, as appropriate. In bivariate analysis, variables with a *p*-value < 0.05 were considered statistically significant and were subsequently included in the multivariable analysis. Binary logistic regression was employed for the multivariate analysis. Given the skewed distribution of nodule size, nodules were categorized into two subgroups (<4 cm and ≥4 cm) for inclusion in the regression model. Statistical analysis was performed using SPSS software, version 25.0 (IBM Corp., Armonk, NY, USA).

## 3. Results

A total of 177 patients met the inclusion criteria for this study. The mean age of the cohort was 55.9 years and the mean body mass index (BMI) was 29.6 kg/m^2^. FNAB results were classified as Bethesda III (AUS) for 153 patients (86.4%) and Bethesda IV for 24 (13.6%) patients. Median nodule size (IQR) was 2.8 cm (1.95–4.0 cm). All patients had benign molecular testing results using the Afirma GEC or GSC.

**Figure 1:**
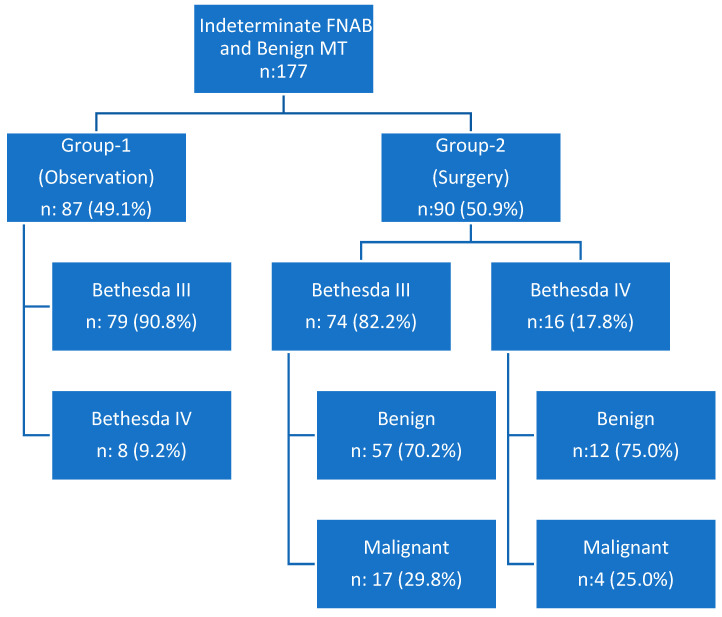
Flow chart represents Patient groups, Fine Needle Aspiration Biopsy and final pathology results. (FNAB: Fine Needle Aspiration Biopsy, MT: Molecular Testing).

Based on clinical decision-making, the observation group (Group 1) included 87 patients (49.1%) and the surgery group (Group 2) included 90 patients (50.9%) (Figure 1). Demographic characteristics, clinical findings, and results of the bivariate analysis comparing the two groups are presented in Table 1.

**Figure 2:**
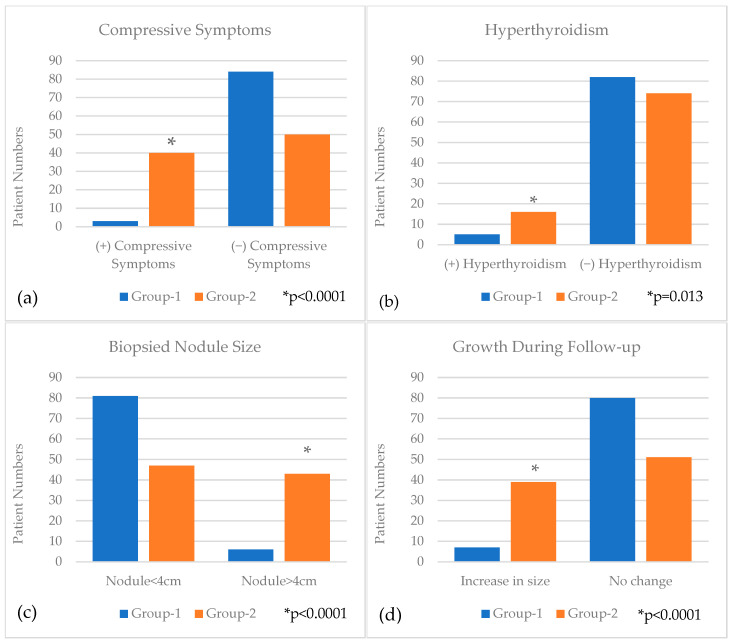
Compressive symptoms (**a**), Hyperthyroidism (**b**), Biopsied Nodule Size (**c**) and Growth During Follow-up (**d**) in comparison between Group-1 and Group-2.

Bivariate analysis demonstrated that the presence of compressive symptoms (*p* < 0.001), hyperthyroidism (*p* = 0.013), nodules >4 cm in size (*p* < 0.001), and documented nodule growth during follow-up (*p* < 0.001) were significantly more common in Group 2 (Figure 2). The variables identified as statistically significant on bivariate analysis were subsequently included in a multivariable logistic regression model. This analysis revealed that patients with compressive symptoms (OR: 23.2; 95%CI: 6.06–88.89; *p* < 0.001), hyperthyroidism (OR: 5.87; 95%CI: 1.63–21.20; *p* = 0.007), nodule size > 4 cm (OR: 11.36; 95%CI: 3.90–33.12; *p* < 0.001), and increasing nodule size during follow-up (OR: 7.85; 95%CI: 2.72–22.65; *p* < 0.001) were independently associated with the decision to proceed with thyroid surgery (Table 2).

In Group 2, total thyroidectomy was performed in 29 patients (32.2%), lobectomy in 58 patients (64.4%) and isthmectomy in 3 patients (3.4%). Final postoperative pathology revealed malignant findings in 21 patients (23.3%) and benign pathology in 69 patients (76.7%). Among the malignant cases, 7 patients (33.3%) were diagnosed with papillary thyroid microcarcinoma (33.3%) and 4 patients (19%) with Noninvasive Follicular Thyroid Neoplasm with Papillary-like Nuclear Features (NIFTP). The remaining 10 patients (47.7%) were diagnosed with either invasive follicular variant papillary thyroid carcinoma, follicular thyroid carcinoma, oncocytic carcinoma of the thyroid (formerly Hurthle cell carcinoma), poorly differentiated thyroid carcinoma or had metastatic disease from another primary malignancy. A detailed summary of final pathology results is presented in Table 3. Only 5 (23.8%) patients required a completion thyroidectomy.

## 4. Discussion

FNAB remains the primary diagnostic tool for evaluating thyroid nodules identified on ultrasound [12,13,14]. It is highly reliable in distinguishing between benign and malignant lesions; however, approximately 30% of the results fall into indeterminate categories, complicating clinical management [5]. Strategies for the management of AUS or FN remain less clearly defined and include options such as active surveillance, repeat biopsy, diagnostic lobectomy or molecular testing, as outlined in the current clinical guidelines [3,15].

Molecular testing, which detects genetic alterations associated with malignancy, has become a widely utilized adjunct in the evaluation of indeterminate thyroid nodules, particularly in the United States [16]. In many clinical settings, cytologic samples obtained during FNAB are preemptively stored and submitted for molecular testing later upon receipt of an indeterminate FNAB result [17]. This practice raises concerns regarding the potential overuse of molecular testing, which may contribute to increased healthcare costs and unnecessary delays in definitive treatment [11,18].

Molecular testing is effective in identifying nodules with a high likelihood of malignancy by evaluating common genetic alterations, such as BRAF and RAS mutations, and gene fusions including PAX8/PPARG, RET/PTC1 and RET/PTC3 [19]. Contemporary molecular platforms report sensitivity of 89–91% and specificity between 68–85% for malignancy [7]. Prior studies have demonstrated the utility of these tests in reducing the frequency of unnecessary surgical interventions for indeterminate nodules. For instance, Jug et al., reported that only 13% of indeterminate nodules with benign molecular testing proceeded to surgery [20]. Similarly, Fung et al., highlighted the potential for molecular testing to reduce the number of unwarranted thyroidectomies [21]. Unfortunately, there is no definitive preoperative molecular test for diagnosing thyroid cancer in indeterminate thyroid nodules. Previous studies have reported false-negative rates of up to 5.8%, which may be associated with increasing nodule size [22,23,24]. In such cases, postoperative pathological evaluation may reveal papillary thyroid carcinoma, follicular thyroid carcinoma, oncocytic carcinoma, or, rarely, poorly differentiated thyroid carcinoma [25,26].

However, much of the literature to date has focused on surgical reduction rates rather than the influence of clinical presentation on decision-making. Steinmetz et al., found that 26% of patients with benign molecular testing still underwent surgery, though their analysis included cases where total thyroidectomy was performed due to a suspicious molecular profile, potentially confounding interpretation [27]. Moreover, because molecular testing results were not blinded to decision-makers in that study, the findings may overestimate the test’s influence on clinical management.

In routine clinical practice, the decision to proceed with thyroidectomy is often based on factors beyond malignancy risk alone [28]. Patients may require surgery for compressive symptoms, hyperthyroidism, concomitant parathyroid disease, or other clinical indications. In our study, 50.9% of the patients with indeterminate FNAB results and benign molecular test results underwent surgical intervention, primarily due to clinical factors. The presence of compressive symptoms was the strongest predictor of surgery, followed by nodule size > 4 cm, nodule growth during follow-up, and hyperthyroidism. These findings suggest that molecular testing offers limited value in altering clinical management in patients who already meet one or more of these criteria for surgery. The only contribution of molecular testing for this patient group lies in guiding the extent of surgery. In patients with thyroid nodules larger than 4 cm and molecular test results suspicious for malignancy, a total thyroidectomy may be considered. Therefore, we do not recommend routine molecular testing in patients presenting with these specific indications unless there is a specific need for information to determine extent of surgery.

Supporting this view, Noureldine et al., retrospectively applied a standardized clinical management protocol and found that molecular test results did not change the surgical decision in 91.6% of cases [11]. In a subsequent prospective study by the same group, molecular testing, influenced surgical decision-making in only 7.9% of the patients [18]. This study also observed a lower frequency of molecular test utilization among patients with compressive symptoms and hyperthyroidism, likely due to the clear need for surgery in these patient groups. Especially in patients with toxic nodules confirmed by thyroid scintigraphy, FNAB, and molecular testing, clinical practice would not change due to the low rates of malignancy [3,29,30]. Our findings reinforce these interpretations that clinical indications often outweigh molecular tests and may not only optimize clinical outcomes but also reduce unnecessary costs and expedite timely surgical care.

Among patients who underwent surgery despite benign molecular testing, 21 patients (23.3%) were found to have malignant pathology, including papillary thyroid microcarcinoma, follicular variant of papillary carcinoma, NIFTP, follicular carcinoma, poorly differentiated thyroid carcinoma and carcinoid tumor. This malignancy rate is consistent with prior studies evaluating outcomes in this patient population [22,31,32]. Pathology results of the 4 patients (19.1%) were consistent with oncocytic carcinoma of the thyroid gland. This ratio is more than expected, however, these results may be attributed to the relatively lower success rate of Afirma testing in detecting oncocytic carcinoma compared to papillary thyroid carcinoma. In the previous studies, a high false positive rate of molecular testing was shown for oncocytic nodules [33,34,35]. Due to the relatively low number of patients who underwent resection of thyroid nodules with benign molecular testing the false negative rate of the molecular testing on oncocytic nodules may not be studied thoroughly. Future studies are needed to understand the rate of false negative molecular testing results for oncocytic thyroid nodules.

This study has several limitations. First, the retrospective nature of this study limits control over potential confounding variables and clinical decision-making processes. Second, the relatively high proportion of patients undergoing surgery may reflect underlying socioeconomic factors, insurance considerations, or institutional surgical preferences. Third, there may be important factors that were not assessed in this study or that were excluded from the medical record.

## 5. Conclusions

A benign molecular test result does not significantly influence the decision to proceed with surgery in patients presenting with compressive symptoms, hyperthyroidism, nodule size > 4 cm or progressive thyroid growth during follow-up. In such cases, clinical indications independently justify surgical intervention. Therefore, the decision to utilize molecular testing should be individualized and guided by a thorough clinical evaluation to ensure cost-effective and timely patient care.

## Figures and Tables

**Table 1:** Demographic and clinical information of the study population. Significant *p*-values were shown as bold and italic. (ACR-TIRADS: American College of Radiology Thyroid Imaging Reporting and Data System, BMI: Body Mass Index, FNAB: Fine Needle Aspiration Biopsy, IQR: Interquartile Range, SD: Standard Deviation).

Bivariate Analysis		All Patients (*n*: 177)	Group-1 (*n*: 87)	Group-2 (*n*: 90)	*p*-Value
Age (years) (mean ± SD)		55.9 ± 13.9	57.2 ± 13.3	54.7 ± 14.6	0.218
Gender	Female	141 (79.7%)	72 (82.8%)	69 (76.7%)	0.314
	Male	36 (20.3%)	15 (17.2%)	21 (23.3%)	
Ethnicity	White	120 (67.8%)	60 (69.0%)	60 (66.7%)	0.583
	Black	52 (29.4%)	26 (29.9%)	26 (28.9%)	
	Asian	4 (2.3%)	1 (1.1%)	3 (3.3%)	
	Hispanic	1 (0.6%)	-	1 (1.1%)	
Preoperative Weight (kg) (median(IQR))		82.9 (70.45–101.3)	83.5 (72.1–100.2)	82.1 (70.3–102.3)	0.840
Preoperative BMI (kg/m^2^) (median(IQR))		29.6 (25.4–36.6)	29.6 (25.7–35.7)	28.9 (25.4–36.9)	0.824
Compressive Symptoms	Yes	43 (24.3%)	3 (3.4%)	40 (44.4%)	***<0.0001***
	No	134 (75.7%)	84 (96.6%)	50 (55.6%)	
Radiation History	Yes	7 (4.0%)	2 (2.3%)	5 (5.6%)	0.444
	No	170 (96.0%)	85 (97.7%)	85 (94.4%)	
Hyperthyroidism	Yes	21 (11.9%)	5 (5.7%)	16 (17.8%)	***0.013***
	No	156 (88.1%)	82 (94.3%)	74 (82.2%)	
Hypothyroidism	Yes	20 (11.3%)	10 (11.5%)	10 (11.1%)	0.936
	No	157 (88.7%)	77 (88.5%)	80 (88.9%)	
Family History thyroid disease	Yes	34 (19.2%)	15 (17.2%)	19 (21.1%)	0.514
	No	143 (80.8%)	72 (82.8%)	71 (78.9%)	
Nodule Size (cm) (median(IQR))		2.8 (1.95–4)	2.2 (1.7–2.9)	3.7 (2.6–4.7)	***<0.0001***
Nodule Location	Isthmus	8 (4.5%)	4 (4.6%)	4 (4.4%)	0.779
	Right	89 (50.3%)	46 (52.9%)	43 (47.8%)	
	Left	80 (45.2%)	37 (42.5%)	43 (47.8%)	
Nodule Size Category	<4 cm	128 (72.3%)	81 (93.1%)	47 (52.2%)	***<0.0001***
	>4 cm	49 (27.7%)	6 (6.9%)	43 (47.8%)	
Number of Thyroid Nodules	Single	76 (42.9%)	41 (47.1%)	35 (38.9%)	0.268
	Multiple	101 (57.1%)	46 (52.9%)	55 (61.1%)	
Growth during Follow-up	Yes	46 (26.0%)	7 (8.0%)	39 (43.3%)	***<0.0001***
	No	131 (74.0%)	80 (92.0%)	51 (56.7%)	
ACR-TIRADS Score	2	2 (1.1%)	-	2 (2.2%)	0.098
	3	63 (35.6%)	25 (28.7%)	38 (42.2%)	
	4	96 (54.2%)	52 (59.8%)	44 (48.9%)	
	5	16 (9.0%)	10 (11.5%)	6 (6.7%)	
FNAB results	Bethesda 3	153 (86.4%)	79 (90.8%)	74 (82.2%)	0.095
	Bethesda 4	24 (13.6%)	8 (9.2%)	16 (17.8%)	

**Table 2:** Multivariable analysis of parameters that may play role in the decision making for thyroid surgery. Significant *p*-values are shown as bold and italic.

**Multivariate Analysis**		**Odds Ratio**	**95% Confidence Interval**	***p*-Value**
Compressive Symptoms	No	Reference		***<0.0001***
	Yes	23.2	6.058–88.887	
Hyperthyroidism	No	Reference		***0.007***
	Yes	5.874	1.628–21.197	
Biopsied Nodule Size	<4 cm	Reference		***<0.0001***
	>4 cm	11.359	3.896–33.117	
Increasing Size during Follow up	No	Reference		***0.0002***
	Yes	7.850	2.721–22.649	

**Table 3:** Postoperative malignant pathology results of the patients in the Group-2.

7 Patients	6 Patient	Incidental Papillary Microcarcinoma, 0.1–0.5 mm
	1 Patient	Papillary Thyroid Microcarcinoma, 7.5 mm
4 patients		Noninvasive follicular thyroid neoplasm with papillary-like nuclear features (NIFTP)
4 patients		Oncocytic carcinoma of the thyroid
3 patients		Follicular thyroid carcinoma
1 patient		Poorly differentiated thyroid carcinoma
1 patient		Carcinoid tumor metastasis
1 patient		Invasive follicular variant papillary thyroid carcinoma

## Data Availability

The raw data supporting the conclusions of this article will be made available by the authors on request.
